# Understanding school frontline workers’ early implementation behavior: insights from the generation healthy kids study in Denmark

**DOI:** 10.3389/fspor.2025.1534123

**Published:** 2025-06-02

**Authors:** Jonas Vestergaard Nielsen, Sofie Koch, Tine Hyldahl Henriksen, Rikke Fredenslund Krølner, Line Lund, Louise Ayoe Sparvath Brautsch, Line Kattai Ulrikkeholm, Camilla Trab Damsgaard, Malte Nejst Larsen, Thomas Skovgaard

**Affiliations:** ^1^ Department of Sports Science and Clinical Biomechanics, University of Southern Denmark, Odense, Denmark; ^2^ National Institute of Public Health, University of Southern Denmark, Copenhagen, Denmark; ^3^ Department of Nutrition, Exercise and Sports, Faculty of Science, University of Copenhagen, Frederiksberg, Denmark

**Keywords:** implementation research, school-based program, qualitative, frontline workers, consolidated framework for implementation research, COM-B

## Abstract

**Introduction:**

Childhood obesity is a growing global public health issue, with significant implications for children’s physical and psychological well-being. Schools offer a structured environment for implementing health-promoting interventions to address this challenge. This study examines how organizational conditions within schools influence frontline workers’ (e.g., teachers and pedagogic staff) early implementation behavior in a school-based health intervention. Insights aim to inform strategies for enhancing the sustainability and effectiveness of such programs.

**Methods:**

The study utilizes a qualitative design, conducting 11 focus group interviews across 11 of the 12 intervention schools participating in the Generation Healthy Kids (GHK) intervention program in Denmark. Using the Consolidated Framework for Implementation Research (CFIR) and the COM-B Model for Behavior Change, the interviews explored organizational factors shaping frontline workers’ capabilities, opportunities, and motivation for implementing GHK components. Thematic analysis, guided by predefined theoretical constructs, was applied to identify key patterns in the data.

**Results:**

The findings highlight organizational conditions within schools as critical to frontline workers’ early implementation behavior. Capability was linked to training and resources, though time constraints hindered effective application. Opportunity was shaped by structural factors such as limited access to facilities, causing logistical challenges. Motivation was influenced by alignment with existing workflows, reflective beliefs about the program’s relevance, and tension for change due to perceived pupil needs. Leadership emerged as a cross-cutting factor, with engaged leaders enhancing implementation through active support and resource coordination.

**Discussion:**

The study underscores the interplay between organizational conditions and frontline workers’ behaviors in implementing health programs. Addressing logistical barriers, fostering supportive leadership, and ensuring alignment with school priorities are essential for successful implementation. These findings highlight the need for adaptive, school-specific implementation strategies to sustain health-promoting interventions like GHK and suggest stakeholder engagement to further optimize implementation processes.

## Introduction

Childhood obesity is a significant public health issue worldwide, posing severe threats to children’s overall well-being and long-term physical health (1–3). The consequences of childhood obesity extend beyond immediate health risks, and obesity is associated with lower psychological well-being, negative social experiences (e.g., bullying and stigmatization), and increases the risk of obesity in adulthood (4–6). In Denmark, the prevalence of overweight and obesity among children aged 6–7 is 12%–13%. This figure increases through elementary school, reaching 18%–19% among children aged 14–15 years (7, 8). This age-related incline highlights the urgent need for early prevention and comprehensive strategies to address the varied factors contributing to childhood obesity. Schools have specifically been identified as a promising setting for health promoting activities due to the marked possibility to reach children of all socioeconomic groups, and because most children spend a large proportion of their everyday in school (9–12). Furthermore, schools provide a structured environment for targeting obesity prevention through interventions like diet and physical activity (13–15).

### The role of frontline workers in early implementation stages

Early implementation refers to the initial phase of program delivery, ideally initiated after an installation phase during which foundational structures, resources, and competency drivers have been organized to support the upcoming implementation activities (16). This early implementation process represents a critical period of adjustment, where intervention activities are introduced into existing organizational routines, and where high expectations is likely to meet resistance to change and entrenched habits within organizations and individuals (16). Michael Lipsky’s street-level bureaucracy theory (17) stands as an early major work on the characteristics and impact of frontline workers such as teacher and pedagogic staff. Lipsky defines frontline workers as actors who interact directly with citizens daily and who make large amounts of discretionary decisions in those relations. In that respect, teachers and pedagogic school staff can be defined as frontline workers. Teachers and pedagogic staff are often designated as responsible for delivering school-based interventions and therefore the success of school-based programs is often highly dependent on their capabilities, willingness and interests in delivering the intervention. This underscores the importance of frontline workers’ implementation behavior, which refers to the actions and decisions of individuals or teams within an organization that directly impact the adoption, adaptation, and execution of an intervention or program in a given context (16). According to Lipsky, the sum of frontline workers’ actions implies that they function as actual policy decision-makers. It is at the end of the implementation chain that policies are realized by welfare professionals such as teachers and pedagogues (17, 18).

### Conditions modeling frontline worker behavior

Although teachers’ cumulative actions significantly influence the realization of school-based interventions and may be seen as a primary agent of change within the school environment, organizational conditions and structural factors play a critical role in shaping their individual implementation behaviors (16, 19, 20). Adequate managerial and leadership support and resources are also crucial in overcoming barriers to implementation—for one thing, because frontline workers are motivated and feel capable of realizing program goals when supported by such stakeholders (21). At the same time, organizational factors within educational settings can significantly influence the success of programs by shaping the context in which frontline workers operate, as well as their behaviors (22). Research has consistently underscored the importance of organizational factors in the successful adoption of educational programs, highlighting how a supportive and structured environment is essential for shaping implementation behaviors (23–25). These findings collectively illustrate the close relationship between organizational conditions and frontline worker behaviors in school-based programs. Additionally, evidence suggests that explicitly linking implementation strategies to the specific organizational contexts of schools increases the likelihood of successful integration and sustainability of new programs (26).

As such, the local organizational conditions help form the behavior of frontline workers. These frontline workers are responsible for delivering the program. Their behavior, in turn, influences whether the intended outcomes related to health and well-being are achieved (27, 28). Frontline workers play a vital role in the initial stages of implementation and throughout the entire process. This highlights the need for studies that focus on multi-component school programs and how these are delivered by frontline workers. Such research is essential to addressing a broader gap in the literature on school-based strategies and their implementation (29). Thus, the present study aims to examine how organizational conditions within the school shape frontline workers’ early implementation behavior. The goal is to generate insights that can contribute to the development of implementation strategies targeting a school setting, and enhancing their adoption, implementation, effectiveness and sustainability.

## Material and methods

### The generation healthy kids study

The Generation Healthy Kids study (GHK) is a comprehensive school intervention program designed to promote healthy weight development among Danish children aged 6–11, running over two school years. GHK is a cluster-randomized trial conducted across 23 schools (12 intervention and 11 control schools) in Denmark (30), containing a multi-setting, multi-component intervention that utilizes a whole-systems approach. Across the intervention schools, located in various regions of Denmark, GHK implements structural and educational components across four domains that influence childhood healthy weight and wellbeing: diet, physical activity, screen media use, and sleep habits. These four behavioral components are individually well-established as critical factors influencing childhood overweight, obesity, and overall health and wellbeing (31–34). In terms of *diet*, a main component is free school lunch provided four days a week. The school lunch is aligned with the national Food-based Dietary Guidelines and the Nordic nutrition recommendations and designed to improve children’s food courage and liking of healthy foods. Teachers and pedagogic staff act as meal hosts, and food literacy exercises are incorporated into classroom activities. The *physical activity* component includes three 40-minute weekly sessions of organized vigorous activity, based on the FIT FIRST 10 concept (35), alongside efforts to promote active play during recess and in after-school settings. To address *screen media use and sleep practices*, teachers and pedagogic staff guide pupils through classroom exercises, encouraging them to reflect on their habits, supplemented with 3 parent workshops delivered by the research team at each school.

To support the delivery and quality of the intervention, GHK applied specific implementation strategies. Each participating school—both intervention and control—was assigned a dedicated project staff member serving as a liaison. Intervention schools received additional support, including resources for upgrading kitchen facilities and inspirational webinars to foster engagement. Project staff also conducted supervisory visits, particularly focus on FIT FIRST and the school lunch components. Furthermore, all participating teachers and pedagogues received comprehensive training to ensure implementing across all four intervention domains (30). In line with broader calls for transparency in implementation research (36), an overview of these implementation support strategies—covering planned actions, responsible actors, target groups, timing, and intended outcomes—is available in the GHK program protocol (30). For additional detail, readers are referred to the protocol’s supporting files, particularly Supplementary Table S3 (*Details of Generation Healthy Kids Intervention Components*) and Supplementary Table S4 (*Completed TIDieR checklist*), which provide structured, component-specific descriptions of implementation content, delivery, and adaptation.

While the primary aim of this paper is to generate broader insights into how organizational conditions within schools influence frontline workers’ early implementation behaviors, these findings can also inform the ongoing implementation and effectiveness of GHK, particularly regarding its potential impact on reducing childhood obesity and promoting pupil well-being (30).

### Theoretical framework

This study applies the Consolidated Framework for Implementation Research (CFIR) (27) and the COM-B Model for Behavior Change as theoretical frameworks (37, 38). CFIR is a comprehensive framework with five key domains including: *Intervention Characteristics*, which examine the specific features of the intervention itself; *Outer Setting*, which considers the external influences on implementation such as policy and community needs; *Inner Setting*, which focuses on the organizational culture, structure, and resources within the implementing site; *Characteristics of Individuals*, which address the knowledge, beliefs, and motivations of individuals involved; and *Implementation process*, which includes planning, engaging, executing, and reflecting on the implementation (27). The framework constitutes an overarching approach, used across the GHK to evaluate its potential for sustainability and scalability (30). The use of CFIR as a conceptual starting point entails a focus on the organizational factors and dynamics that shape the potential for GHK’s implementation. Given the aim of this study, we specifically focus on the Inner Setting dimension of CFIR to explore how organizational factors within schools’ shape frontline workers’ implementation behavior of GHK. By concentrating on internal organizational culture, structures, and available resources, we aim to gain deeper insights into the ways in which these inner setting elements affect frontline workers’ engagement with GHK. To support this exploration, we integrate the COM-B model’s behavioral lens to examine how these inner settings influence frontline workers’ *capabilities*, *opportunities*, and *motivation*, ultimately shaping their implementation behavior. COM-B posits that *Behavior* is driven by the interaction of *Capability*, *Opportunity*, and *Motivation* (38). Frontline workers’ *capabilities* refer to their knowledge and skills to deliver the program effectively. *Opportunities* pertain to the external conditions that facilitate or hinder the implementation process, such as availability of resources. Finally, *motivation* encompasses the internal drives and incentives that encourage teachers to engage actively with the program while also concerning whether a given behavior becomes an automatic and natural part of a given behavior. Such an in-depth exploration of frontline workers’ capabilities, opportunities, and motivation regarding the implementation of programs like GHK is important for understanding the factors contributing to both immediate and long-term program outcomes.

### Study design and participants

The present study utilizes a qualitative approach to gain a broad understanding of the role of frontline workers in the early implementation of the GHK program. The study employs focus group interviews to explore the experiences and perceptions of frontline workers from the intervention schools—including teachers and pedagogic staff. Participants were recruited across the 12 intervention schools. The 12 schools represent both public (*n* = 10) and private (*n* = 2) institutions. Schools were distributed across urban (*n* = 7) and rural (*n* = 5) locations and varied in size, with student populations ranging from 173–778 pupils. Further information on participating schools can be found in Thomsen et al. (30). Due to the recruitment strategy for the GHK study (30), a diverse selection of schools in terms of sociodemographic composition, geographic location, size and community context, was represented.

### Data collection

Focus group interviews were conducted to gain in-depth understanding of the experiences of frontline workers, with the aim of capturing their perspectives on implementing GHK components (39). The focus group design allowed for the exploration of nuances and complexities inherent in the early implementation process, providing insight into the diverse experiences, thoughts, and attitudes of frontline workers regarding GHK. A semi-structured interview guide, based on the inner setting domain of CFIR and COM-B, was developed to both facilitate discussions and ensure that all relevant domains and factors within these frameworks were covered (see Supplementary Appendix 1: Interview guide). This semi-structured approach also allowed for openness to responses and insights that extended beyond the predefined themes of the theories. By using open-ended questions, the interview guide supported in-depth, reflective responses, allowing participants to explore their own experiences and perceptions in detail. Participants were also encouraged to comment on each other’s statements, fostering a more dynamic and interactive discussion. The interviews were conducted in the spring of 2024, i.e., 1.5–3 months after the implementation had begun at the schools for FIT FIRST and school lunch, corresponding to the post-installation phase (16), during which the necessary structural supports and competency drivers for program initiation had already been established. At the time of the interviews, the screen and sleep intervention components were still in the installation phase (16), with foundational structures and supports being organized to prepare for their implementation.

Out of the 12 intervention schools, one small school opted not to participate, citing concerns about feeling overwhelmed by the presence of participant observers and testing activities related to GHK. Consequently, they prioritized using teachers’ working hours to maintain the quality of program implementation and focus on everyday core tasks, such as teaching. Thus, a total of 11 focus group interviews were conducted, with one focus group held at each of the remaining 11 intervention schools. Each focus group was comprised of three to six teachers and pedagogic staff who were directly involved in the implementation of the physical activity, diet, or screen and/or sleep aspects of the GHK program. There was a relatively equal distribution of participants involved in the diet and FIT FIRST (physical activity) components. However, participants addressed the screen and sleep components to a lesser extent, as these had not yet been fully implemented in the schools at the time of the interviews. These individuals were recruited by a school contact designated to assist in connecting the GHK project group and involved schools. Interviews were conducted by two GHK project staff members and three master’s students at the schools and arranged to accommodate the informants’ scheduling issues. Interviews lasted between 30 and 50 min.

Prior to data collection, to minimize interviewer bias and ensure a cohesive data collection process, all interviewers participated in preparatory training sessions designed to calibrate their interviewing techniques, streamline their approach, and ensure consistent procedures (40).

### Data analysis

Interviews were transcribed verbatim and pseudonymized prior to analysis. Guided by Braun and Clarke’s (41) framework for thematic analysis (41), the first author conducted a systematic data analysis. The initial phase involved familiarization with the data through repeated reading and immersion to develop a comprehensive understanding of the dataset. Coding was primarily deductive, informed by predefined constructs from the CFIR framework and the COM-B model. An initial codebook was developed before the main coding phase and tested on a single interview. This pilot coding led to minor refinements of code definitions to ensure specificity and alignment with the intended constructs. Using the finalized codebook, a broad initial coding of the entire dataset was conducted, followed by a more detailed, fine-grained coding phase to ensure that each code strictly captured relevant information linked to its corresponding construct. As the coding was conducted solely by the first author, reliability was strengthened through ongoing reflexive engagement with the data, the frameworks, and the emerging analytical interpretations. Throughout the analysis, particular attention was paid to the co-occurrence of codes within the same transcript segments and to thematic proximity across participants’ narratives. Codes were examined not only individually but also for how they clustered together, providing insight into relationships between organizational conditions and frontline workers’ early implementation behavior. To systematically track and refine these relationships, matrices were developed to link codes associated with the COM-B components (Capability, Opportunity, Motivation) and relevant CFIR constructs, ensuring that thematic linkages were grounded in consistent patterns across multiple cases.

The analytical process thus focused on understanding how structural and organizational factors within schools shaped frontline workers’ early implementation behavior in GHK. The analysis identified three key components of the COM-B model (*Capability, Opportunity*, and *Motivation*) alongside the significant cross-cutting influence of leadership, all closely tied to constructs within CFIR. *Capability* was linked with CFIR’s construct of *access to knowledge and information* within the organization and addresses the extent to which frontline workers have the resources and training needed for successful implementation. *Opportunity* was linked with CFIR’s construct of *structural characteristics* and *available resources**,*** which pertains to the tangible assets required for effective implementation. *Motivation* was linked with CFIR’s constructs of *tension for change* and *compatibility*, which focus on the perceived necessity of the program and its alignment with existing values and workflows. Finally, leadership emerged as a cross-cutting influence on all three components of the COM-B model, linked to the CFIR construct of *work infrastructure*. *Work infrastructure* is identified as tasks and responsibilities within and between individuals and teams in the organization—also including leadership through organizing work structures, delegating tasks, and managing resources.

### Ethical considerations

Written informed consent was obtained from all participants prior to their involvement in the study, ensuring they were fully aware of the study’s aims, procedures, and their right to withdraw at any time. All data were anonymized to protect participant privacy and confidentiality and were securely stored in accordance with the General Data Protection Regulation (GDPR) in Europe.

## Results

The findings from the focus group interviews revealed several key themes within the inner setting of the schools, influencing frontline workers’ early implementation of GHK. These were mainly structured around the components of the COM-B model and are closely linked to the organizational constructs within the ‘Inner Settings’ domain of CFIR.

### Capability (access to knowledge and information)

In general, frontline workers with a backgrounds such as Physical Education teaching expressed strong confidence in their capability in delivering the FIT FIRST physical activity component of GHK. Across components, participants generally reported that they felt equipped with the necessary skills after attending the preparatory courses. As one frontline worker explained:

I attended the course on diet and also the FIT FIRST part, and I think it’s a course that really brings everything down to earth. It becomes so tangible and easy to approach [School 10]

Despite these positive experience, some participants still found it challenging to translate the course content into their daily teaching routines. As one noted:

“We need more training to fully understand how to integrate these activities into our daily routine.” [School 4]

This suggest variation in perceived readiness to implement, depending not only on the quality of course activities and materials, but also on the extend to which the material were adapted to the school context and supported daily integration. Importantly, some participants also pointed to a perceived loss of professional autonomy in structuring their schedules and lessons (e.g., to adhere to the needs of their pupils). As a participant highlighted:

There are many things we have to take into account, and there are also some things we no longer do because of this—shorter lessons due to the meals [red. the free school lunch delivered as part of GHK] and fewer things we can decide for ourselves, as much of it is predetermined in relation to physical activity. [School 8]

Such experiences reflect tensions between standardized program content and local pedagogical judgment. In several cases, these tensions were linked to frustrations around scheduling and prioritization, as participants struggled to fit new activities into existing demands:

It’s hard to fit these new activities into our schedule, and sometimes it feels like we’re sacrificing core subjects. [School 2]

Because we’re all fighting, the teachers among us, to get our hours so we can cover our curriculum. [School 5]

This frustration reflects the tension between adopting a new program and meeting the regular educational demands. Thus, while the project group supported the capability of frontline workers through training and resources, addressing the logistical challenges—in relation to existing priorities and tasks—is equally important to ensure successful implementation of the GHK program.

### Opportunity (structural characteristics and available resources)

Frontline workers reported several barriers related to opportunity, particularly in terms of time, space and facilities. While many felt capable and motivated to deliver the intervention, their ability to do so was often constrained by structural and contextual factors beyond their control.

A frequently mentioned challenge concerned the availability of time to engage meaningfully with the training materials provided and integrate new practices into daily routines. Although participants generally appreciated the easy access to knowledge and information from courses (such as written and visual guides), they expressed frustration over the lack of time to explore and apply them effectively. As participant noted:

I think we got a lot of material. It’s not that we're missing anything…it's more about finding the time to immerse ourselves in it so that we can translate it into something we can use. [School 6]

Time constraints were a recurring theme, indicating that even with adequate knowledge and motivation, the opportunity to implement depended on frontline workers’ ability to prioritize and protect time within a packed school schedule.

Beyond time, several participants highlighted difficulties related to physical infrastructure. Limited access to gyms or suitable spaces posed barriers to implementing the physical activity component of GHK. One frontline worker shared the challenge of negotiating gym space:

Our gym is often booked, and we struggle to find time and space for additional physical activities. [School 1]

Interviews reveal that the struggle to secure adequate facilities resulted in logistical challenges, limiting the ability of frontline workers to fully implement the physical activity sessions. Similarly, for those responsible for the diet component, access to proper kitchen facilities showed some initial difficulties. As one participant mentioned:

The food authorities couldn’t approve our kitchen for use, so we had to move to another location where they’re setting up a new kitchen. [School 11]

This highlights how the physical infrastructure at some schools could act as a limiting factor for the successful implementation of certain program components. Although most of the participants were confident that over time they would find a way to implement GHK at their school, the limited availability of suitable spaces for physical activities, in some schools not only delayed program implementation but also forced frontline workers into complex negotiations over shared school resources, as highlighted by one frontline worker:

We also need the gym or the sports-hall-space, and we have to negotiate with the other teachers and the administration on where we can be. [School 6]

Overall, the findings suggest that logistical constraints—including time, space, and infrastructure—shaped the opportunity to implement the GHK program. This was mostly related to aspects of the physical activity component, as it to a higher degree collided with other school and educational activities (e.g., other classes having physical education lessons). Interviews showed that frontline workers at schools with insufficient or inappropriate facilities were urged to try and find workarounds, which then added to the complexity of implementing the program.

### Motivation (tension for change and compatibility)

Frontline workers’ motivation to implement GHK was strongly influenced by their reflective beliefs about the program’s relevance. Many participants expressed that their motivation depended on their personal commitment to the program’s goals. As one teacher noted,

I believe in the importance of this health program, but it’s challenging to maintain motivation without seeing immediate results. [School 2]

This underscores the need for visible outcomes to sustain long-term motivation, especially when the program demands significant changes to established routines. Teacher motivation also depends on their belief in the program to make a relevant difference and how well it fits to the existing duties and curriculum. At one of the schools, a participant expressed how the physical education teachers find that the GHK physical activity element disrupts their existing structure in delivering the curriculum, as several schools opted to integrate FIT FIRST as part of their physical education:

What they say [the physical education teachers at the school] is that they feel it [FIT FIRST] disrupts their physical education lessons, and that’s why they continue with their own teaching. [School 7]

GHK did not mandate that the physical activity should be conducted during physical education lessons. For instance, some schools maintained an additional weekly double lesson in physical education alongside the three days with the FIT FIRST physical activities, demonstrating a flexible approach to scheduling. However, a small number of partisipants still highlighted this disruption, and that teaching staff did not make efforts to incorporate the GHK components.

The interviews also show a broad belief that GHK addresses real and pressing issues, thus reinforcing frontline workers’ motivation to participate in the program through a *tension for change*. Two nts from different schools highlighted a need for the program in addressing pressing health concerns for their pupils:

I think we have many children who are inactive, use too much screen time, sleep too little, and eat poorly. So, I see this as a huge win-win, especially when parents get educated. Even if it doesn’t fully register with them, the children still get something out of it. [School 3]

As my colleague mentioned, with those kids who may not bring the best lunch, at least we know there’s one good meal during the school day. And the fact that there’s mandatory physical activity three times a week is also really valuable for most of them. [School 9]

According to participants, these accounts reflect not only general health challenges but also perceived social and health disparities within the pupil population, particularly related to diet, activity levels, and access to healthy routines. This also resonates with an acknowledged diversity in the pupil backgrounds, expressing relief that e.g., the school’s lunch scheme addresses inequalities by providing healthy meals for pupils who might not otherwise have access to them:

There’s a lot of diversity among pupils. I also have some kids where I think “thank goodness we have the lunch scheme”. [School 11]

Another frontline worker discussed how their team worked together to ensure that the program’s activities aligned with their schedules and responsibilities:

We also contributed and said it made sense, and then we agreed as a team. We all contributed and agreed that it made sense. Then, as a team, we worked out who could do what and how to fit it all together. [School 1]

These findings suggest that frontline workers’ motivation can be closely tied to how well the GHK program aligns with their existing workflows and the extent to which they are involved in the planning and adaptation of activities.

In summary, *motivation* to implement GHK was driven by a combination of personal commitment to health promotion, the perceived urgency of addressing pupil health disparities, and the early involvement of teachers and other staff in the decision to become part of GHK.

### Work infrastructure and leadership as a cross-cutting influence

The engagement, support, and communication from school leaders were consistently reported as critical to enable frontline workers effective implementation. The findings revealed that most school leaders were actively engaged, and visibly supporting frontline workers—positively influencing their motivation and capability to implement the program. As one participant noted,

There’s been a belief from the school leaders that we could do it, and I think they [the leaders] have contributed to that by being responsive to any challenges we’ve had. [School 10]

At one school, participants also pointed out the crucial role that their school leader played in navigating challenges associated with introducing GHK to the other teaching staff, including logistical challenges—such as ensuring access to spaces and resources. Two participants from different schools elaborated on this:

The other teachers already felt like it was just another project being forced on them… At least some of them did… So, it was really important that we had the backing of the school leader, and we’ve had that so far. [School 11]

We have to negotiate where we can be [for activities], but the leadership has been helpful in coordinating and supporting us to ensure we have the resources we need. [School 4]

This highlights the essential role of leaders in mitigating resistance to change by showing a clear and useful path that supports the early implementation of GHK and helps foster buy-in from frontline workers. When school leadership demonstrated commitment to the program and took on the responsibility of coordinating, it alleviated some of the burden from frontline workers, making them feel supported and less overwhelmed by the introduction of GHK. Moreover, frontline workers reported that the leadership’s involvement went beyond simple oversight and opinion setter, as leaders often took an active role in concrete program planning, which enhanced the overall readiness of the school to implement GHK. One participant mentioned,

The school leadership showed their engagement by taking on the overall coordination. It really helped us because we knew there was someone to turn to if issues arose, and it showed that this program was a priority for the school. [School 1]

This hands-on approach from leaders ensured that GHK became an integral part of the school’s broader agenda, thereby reinforcing its importance among school staff in general.

Several of the participants reflected on the significance of school leadership in facilitating a positive implementation environment. Leadership’s ability to engage, coordinate, and communicate effectively was repeatedly identified as key to both the success of the GHK program and the motivation of frontline workers.

Leadership engagement has been key to ensuring we have the resources we need, whether it’s access to materials or the time to actually use them. Without their involvement, it would be much harder to get everything in place. [School 9]

In summary, participants across the eleven schools highlighted that engaged leadership is a pivotal factor supporting frontline workers in their early implementation efforts. Leaders who took on active roles in coordination, promoted timely, inclusive and accurate program communication, and prioritized the program within the school created an environment enhancing frontline workers sense of organizational support and empowerment. This suggests that leadership not only provided the structural and logistical resources needed but also were important in fostering a positive organizational culture that encouraged engagement with the program. This proposes that involvement, support, responsibility, backing, and coordination from school leaders are important factors that support program realization through structural and logistical resources while also nurturing an organizational culture that contribute to frontline workers feeling capable of and motivated to implementing GHK.

## Discussion

The findings offer insights into the factors that shape frontline workers early implementation process. Utilizing the COM-B model and CFIR framework, the analysis reveals that successful implementation is influenced by a complex interplay of capability, opportunity, and motivation. These components are affected by both the characteristics of the frontline workers and the organizational context, particularly the role of leadership. The results underscore the need for adequate time, availability of facilities, and strong leadership to optimize the implementation of school-based health programs like GHK. These aspects are discussed further in the following.

### Frontline workers at the core

Results show that frontline workers had a perceived tension for change, through a recognition that the current situation concerning their pupil’s health behavior (e.g., unhealthy lunch boxes or screentime habits) compels change. This concept is related to, but not the same, as readiness for Change, which can be defined as the degree to which stakeholders accept, embrace, and adopt a specific plan to change the status quo (28). Despite a perceived tension for change, frontline workers, such as teachers, can still face a “battle of values and resources” in deciding which priorities to address—whether to allocate time to core subjects like mathematics or to focus on food, physical activity, and health-related programs (18, 42, 43). Teachers and school staff often face competing demands, including curriculum requirements, administrative expectations, and the diverse needs of pupils (29, 43). This prioritization of tasks and resources was also evident during the recruitment for interviews, as one school opted to use teachers’ working hours to maintain the quality of program implementation and focus on everyday core tasks, such as teaching. In GHK, this tension was further highlighted by several participants who expressed the challenge of integrating health-focused activities into already packed school schedules, which led to concerns about sacrificing core subjects. These findings are further supported by literature highlighting the challenge of balancing intervention demands with existing educational priorities, emphasizing the need for consistent evaluation of implementation contexts, organizational readiness, resources, and leadership as critical barriers to successful program implementation (25, 44).

Programs that align well with existing organizational and/or professional values are more likely to be embraced and implemented with fidelity (29, 43, 45). A key element of Lipsky’s theory is recognizing that frontline workers operate under constraints, such as limited time, resources, and institutional support (18), a point that has been further substantiated by subsequent studies highlighting similar challenges in various contexts (15, 16, 21). This was reflected in the findings, where teachers frequently mentioned the lack of time and facilities as significant barriers to implementing GHK effectively. As Lipsky (17) suggests, these constraints force frontline workers to make choices about what aspects of the program to prioritize, which can result in variations in the quality and fidelity of implementation across different schools.

### From individual engagement to organizational embedding

The findings suggest that frontline workers’ individual engagement can have a significant impact on programs like GHK, as their decisions and behaviors shape the organizational culture that these programs aim to transform (18). Their participation is crucial in embedding long-lasting, sustainable health practices within the school environment. Thus, the success of GHK very much depends on frontline workers’ buy-in with the program’s goals and their ability to navigate these competing demands. Findings revealed that during the initial stages of the implementation, teachers often expressed a need for more time to engage with the program’s materials, which posed a barrier to an extensive implementation.

This challenge is further compounded by the goal to, if not alter, then adjust established school cultures, finely balanced by existing tasks and available resources (46, 47). This implies that implementing a new practice in school settings necessitates organizational change, and programs must be flexible enough to ensure sufficient local adaptability to accommodate teachers’ professional autonomy, and provide directly usable resources (knowledge, facilities, tools, manpower etc.) (48, 47). Such goals and premises bring with it the possibility, or more likely, the probability, of individuals and groups questioning, or at least considering, the relative advantages of changes to be implemented and, at the same time, showing a preference to hold on to the status quo (49, 50).

In relation to CFIR, the concept of *relative priority* becomes particularly relevant (27). The results suggest that the priority given to GHK by school leaders and teachers influenced its early implementation. When teachers and other frontline workers perceive the implementation of a new program as a high organizational priority—backed by institutional support and reward systems—the implementation climate becomes more conducive to success (21, 51). However, if teachers are overwhelmed by multiple competing programs, the program risks being deprioritized or seen as a distraction from their core tasks (52). This tension closely mirrors Lipsky’s notion of competing values, where teachers must balance the demands of delivering core academic content with the objectives of programs like GHK (18). Some participants reported that the GHK activities were seen as disruptive by their colleagues, especially among physical education teachers who felt their regular teaching schedules were being interrupted. In this context, ensuring that GHK is viewed as a central priority is pivotal to fostering motivation and engagement among frontline workers, increasing the likelihood of achieving the program’s intended outcomes.

As an example, the findings reveal that leadership engagement extended beyond merely providing resources and general support. Participants noted that leaders played an active role in alleviating resistance to change by framing the program as a high organizational priority, which was essential in fostering teacher buy-in. As highlighted by frontline workers, when leadership figures demonstrated active support, it allowed teachers to feel less overwhelmed by the new responsibilities associated with GHK. Furthermore, the findings suggest that while frontline workers’ initial capabilities and motivation are crucial, ongoing support and adaptive leadership are equally important for sustaining implementation efforts. This underscores the critical role of frontline worker motivation and engagement in shaping the organizational culture aimed at by programs like GHK. Teachers’ initial capabilities and motivation are essential, yet ongoing organizational support and adaptive leadership are equally crucial for sustaining implementation efforts (24, 43, 53).

### Leaders frame the inner setting

Despite small variations across the schools, frontline workers generally reported that they had the necessary skills, opportunities, and motivation to deliver the GHK program. However, a range of factors—including school leaders’ engagement, coordination, and prioritization of the GHK program impacted the success of implementation. This aligns with the argument that explicitly linking implementation strategies to the organizational context enhances the likelihood of successful and sustainable interventions, as well as the notion that leadership plays a crucial role in creating a supportive implementation climate conducive to adherence to guidelines (24, 26).

Our findings further indicate that leadership behavior significantly influence the organizational climate and readiness for change (54, 55). Therefore, enhancing leadership capabilities through targeted training and strategic support can strengthen the sustainability and effectiveness of interventions like GHK (25).

The findings highlight the determining role of leadership in the successful implementation of school-based programs like GHK. If leadership engagement in fact is crucial, there is a strong case for further investment in supporting school leaders throughout the implementation process. This suggests the need for upskilling leaders and equipping them with the tools necessary to facilitate complex program effectively. Future research should explore strategic opportunities and develop frameworks to support school leadership in processes such as these. By strengthening the capacity of leaders, the sustainability and long-term impact of programs like GHK are more effectively realized, particularly in diverse school contexts where leadership frequently proves crucial.

### Relational connections in the implementation setting

The implementation of large-scale, multi-component programs requires not only changes at the individual level but also significant organizational shifts and prioritization across the school. This type of program extends beyond the daily routines of the teachers and pupils directly involved, influencing other branches of the school’s organizational structure. This is evident in the challenges related to finding time and physical facilities, and the negotiations that arise when a project as comprehensive as GHK is introduced. Such organizational adjustments emphasize the interconnectedness of the entire school environment, where also those not directly involved in a given program might be affected.

This point touches on an issue raised in the literature, regarding the role of team dynamics in ensuring successful program implementation. Studies have highlighted that a sense of *relational connections* or community among staff contributes significantly to implementation outcomes (27, 56, 57). CFIR emphasizes the development of cohesive teams to strengthen the collective capacity to engage with complex programs like GHK. Within GHK, some participants noted how team cooperation was vital for overcoming logistical challenges, as one teacher expressed how they worked together to balance schedules and responsibilities. This finding reinforces the importance of relational connections, where cohesive team structures allow for collective problem-solving and support, which supports the notion of how team-level implementation outcomes relates to the successful program implementation (58).

Finally, it should be highlighted that every organization operates within a formal structure, which offers both constraints and opportunities (59). Therefore, it is essential for research-based programs like GHK to understand how each school works as an organization, and how to use this understanding to foster productive collaboration, ultimately leading to more effective partnerships (60, 61). Fostering strong organizational partnerships between schools and the GHK program is crucial for long-term impact, and without clear strategies and structured communication channels, frontline workers may deprioritize such programs, seeing them as secondary to their core teaching responsibilities. In the context of the GHK program, individual school contacts and communication channels have been established to promote effective collaboration of program delivery and data collection (30). To our knowledge, however, detailed strategies and guidelines on how to establish such effective communication and collaboration between research programs like GHK and schools have not been developed, warranting further investigation in future studies. Ultimately, the success of school-based health programs like GHK depends not only on effective organizational partnerships but also on the personal engagement of teachers within the school environment.

### Limitations and future research

While the study provides valuable insights, it is not without limitations. One being the reliance on self-reported data from frontline workers, which may be subject to social desirability bias or may not fully capture the complexities of the implementation process (62). Additionally, the focus on a specific program within a particular cultural context may limit the generalizability of the findings to other settings or populations. The study’s cross-sectional design also does not allow for an examination of how the implementation processes and outcomes evolve over time, which could provide a deeper understanding of the factors influencing sustained implementation. Furthermore, fidelity to the intervention components has not yet been systematically assessed or reported. These data are planned as part of the broader GHK evaluation (30) but are not yet available.

An important and unanswered question in the current literature is whether and how a “tipping point” can be reached where the attitudes and buy-in of school staff is predominantly supportive of a research driven program (43). Future research should establish longitudinal studies that could provide a deeper understanding of how frontline workers’ roles and the factors influencing them evolve over time. Additionally, investigating how frontline workers’ perceptions and practices change over time could also provide more insights into the long-term sustainability of school-based health programs.

Expanding the scope to include multiple stakeholders, such as pupils, parents, and external partners, could offer a more holistic view of the factors influencing the implementation of health programs in schools. Such stakeholder perspectives are planned to be explored in the GHK, but not yet published (30). Moreover, further research examining the relationship between frontline workers’ motivation, engagement, and the quality of implementation is essential for identifying strategies that improve program outcomes.

Finaly, the integration of the COM-B model and CFIR in this study offers a notable effort in the further theoretical development of implementation science. By demonstrating the utility of combining behavioral and implementation science frameworks, the study explores a new approach for examining the complex interplay between individual behaviors and organizational contexts. This integration suggests that future studies could benefit from employing multiple theoretical perspectives to explore the various dimensions of implementation processes.

### Implications and future directions

The study’s findings contribute to a refined understanding of the role of frontline workers in earlyimplementation, emphasizing the importance of considering both individual and contextual factors. This aligns with the growing recognition within implementation science that successful programs require both the capacity for change at the individual level and an environment that supports and sustains those changes over time.

Specifically, the study offers several contributions to the field of school-based health program implementation. First, it provides early-stage insight into how frontline workers experience and respond to a complex, multi-component program in a real-world school context. Second, by combining the COM-B model and CFIR framework, the study contributes to a more integrated understanding of how individual-level motivation, capabilities, and contextual opportunities interact during the initial phase of implementation. Third, it points to the importance of leadership and organizational conditions—such as time constraints, professional autonomy, and resource availability—not as passive background elements but as active influences on implementation behavior. Finally, the study draws attention to relational and structural aspects of implementation, including team-level coordination and organizational readiness, which may be particularly relevant in schools facing diverse and demanding circumstances. These findings complement existing research by offering a practice-oriented perspective on the micro-level dynamics of early implementation.

In conclusion, this study highlights the crucial role of frontline workers in the implementation of the GHK and provides insights into the factors that influence successful implementation. By moving beyond general barriers and facilitators, the study foregrounds the situated experiences of frontline workers navigating the complex interplay between motivation, leadership, and organizational context. By integrating behavioral and implementation science frameworks, the study advances our understanding of the dynamic and complex nature of implementation processes in school settings. Future research should continue to build on these findings to further refine theoretical framework and models and develop practical strategies for improving the implementation of health-related programs in schools. Longitudinal designs and multi-stakeholder perspectives may be particularly valuable for capturing how implementation evolves and becomes embedded over time.

## Data Availability

The datasets presented in this article are not readily available because due to legal and privacy issues concerning the European General Data Protection Regulation (GDPR). Requests to access the datasets should be directed to the second author Sofie Koch (skoch@health.sdu.dk).
