# Supplementary material for: Understanding school frontline workers’ early implementation behavior: insights from the generation healthy kids study in Denmark
Source: Front Sports Act Living. 2025 Jun 2;7:1534123. doi: 10.3389/fspor.2025.1534123 (PMC12171354; doi:10.3389/fspor.2025.1534123)
Supplement: Supplementary file 1 [file Table1.docx]

# **Appendix 1 - Interview Guide**

# **Introductory questions:**

# *Theory: CFIR Inner Setting*

# Can you start by sharing how each of you is involved in the Generation Healthy Kids initiative?

# How did you get your roles in this project at the school (were you asked, and if so, by whom? Did you volunteer on your own initiative?)?

# How is the coordination of Generation Healthy Kids activities carried out at the school?

# Is coordination something you do collectively, or does it happen more within each of the four focus areas in Generation Healthy Kids (physical activity, diet, sleep, and screen time)?

# **Frontline Workers – Delivery of the Intervention:**

# *Theory: CFIR Inner setting and COM-B*

# What are your general thoughts on the Generation Healthy Kids initiative?

# Are the FIT FIRST sessions easy to conduct for you?

# Do the lunch program and the general focus on food interest the students? And are you satisfied with your role in this?

# To what extent do you feel that the Generation Healthy Kids initiative fits into the daily life of the school?

# To what extent do you find the Generation Healthy Kids initiative to be a relevant initiative for your school?

# Are there parts you find more relevant than others?

# Have you previously focused on one or more of the themes that are the focus of Generation Healthy Kids?

# To what extent do you find it possible to deliver what each of you is responsible for in Generation Healthy Kids here at the school?

# How do you perceive the engagement for Generation Healthy Kids here at the school? Among your colleagues, leaders, students, and parents?

# Can you give some examples of some activities where you experience particular engagement from students/parents or your colleagues?

# In which activities do you feel your own engagement is greatest?

# To what extent do you feel equipped to handle the tasks you are responsible for in Generation Healthy Kids at your school?

# Are there any information or materials you feel you lack? If so, how has this affected your work with Generation Healthy Kids?

# Who do you go to if you have questions related to Generation Healthy Kids?

# What challenges do you experience with delivering Generation Healthy Kids at your school (e.g., practical, financial, time-related, professional)?

# What opportunities do you see in working with Generation Healthy Kids that it offers you as employees? (skills development, increased focus on something you have a particular interest in, etc.)

# Are there structural conditions, such as facilities at the school, that have affected your work with Generation Healthy Kids?

# Can you provide specific examples of how these conditions affect your work (positively and/or negatively) with Generation Healthy Kids?

# Do you continuously evaluate how Generation Healthy Kids is going here at the school?

# Do you evaluate via your class teams or across the two grades?

# Who is responsible for the ongoing evaluation?

# **‘Champions’ – What does this role look like at individual schools:**

# *Theory:* *CFIR Inner Setting*

# Do you have a person/people at the school who handles the overall coordination of Generation Healthy Kids?

# Who is this person?

# What is their task(s) in Generation Healthy Kids?

# Do you know how this person got this task? (self-chosen, was asked – and by whom)

# How does having such a coordinator at the school affect your work with Generation Healthy Kids?

# What would happen if the person were not there?

# Can you provide specific examples of when and how your coordinator particularly plays a role for you in relation to Generation Healthy Kids?

# **Staff's View on Leadership in the Implementation of Generation Healthy Kids:**

# *Theory:* *CFIR Inner Setting; Implementation Process****;*** *and Characteristics of Individuals*

# How important do you feel the school leadership is in making a project like Generation Healthy Kids successful?

# What do we mean by school leadership in this context?

# What role does the leadership play in the daily context in relation to Generation Healthy Kids?

# When do you find that leadership plays a particularly important role in making Generation Healthy Kids successful?

# How do you find that your immediate management (e.g., department head) impacts your daily work with Generation Healthy Kids?

# What role does your immediate leader play daily in relation to Generation Healthy Kids?

# Can you give some examples of specific actions or strategies from your immediate leader that have influenced your daily work with Generation Healthy Kids?

# Are there specific initiatives from the leadership side that you would have wished for in relation to your work with Generation Healthy Kids?

# Both with a view to where you are now and also when you look back to the start of Generation Healthy Kids

# What would you consider the most important qualities or attributes of a school leader/school leadership to make a project like Generation Healthy Kids successful?

# And what are the most important qualities or attributes of a department leader or similar to make a project like Generation Healthy Kids successful?

# Are there other actors on/around the school that affect your engagement and implementation of Generation Healthy Kids?

# Do you have collaborations with the local associations or others in your local area?

# Who are these actors?
